# The intention to adopt mental mHealth services in emergencies: pre-engagement social determinants of PTSD-Coach app use

**DOI:** 10.3389/fdgth.2026.1737779

**Published:** 2026-01-20

**Authors:** Keren Mazuz

**Affiliations:** School of Management, Jerusalem Multidisciplinary College, Jerusalem, Israel

**Keywords:** collective trauma, community resilience, digital mental health, help-seeking, intention to adopt, PTSD-Coach, public mental health, self-efficacy

## Abstract

Trauma-focused mobile health (mHealth) applications, such as PTSD-Coach, hold significant potential to address acute psychological needs following large-scale emergencies, yet adoption remains inconsistent. This study examined associations between psychosocial resources and intention to adopt the Hebrew version of PTSD-Coach in Israel after the October 7, 2023, terror attack, which triggered widespread collective trauma and ongoing war. Survey data from Israeli adults (*n* = 86) measured trauma literacy, self-efficacy, citizenship (willingness to share/recommend), and adoption intention. Quantitative analyses using multivariable regression identified a sequential pathway: trauma literacy enabled users to recognize symptom relevance, self-efficacy converted knowledge into capability, and citizenship extended adoption intentions into social spaces. Trauma literacy was the only significant predictor of intention in the full model, while demographic and clinical variables including trauma exposure were non-significant. Self-efficacy strongly predicted willingness to recommend the app, and once self-efficacy was included, the direct effect of knowledge diminished, supporting a sequential process: Knowledge → Self-efficacy → Citizenshi*p* → Intention. Rooted in social psychiatry and trauma-informed public mental health perspectives, this study theoretically interprets how individual psychological resources and social dynamics may shape early digital help-seeking in crisis conditions. Findings suggest that trauma literacy and perceived capability are central correlates of adoption readiness, challenging assumptions that lived trauma experience automatically increases help-seeking. This pattern may reflect how acute stress impairs information uptake and perceived self-efficacy. From a mental health systems perspective, these findings point to the potential importance of proactive psychoeducation, stigma-reduction strategies, and community-based outreach to support digital intervention uptake during collective trauma. Strengthening trauma literacy and self-efficacy may support timely self-management, help-seeking, and community resilience where formal psychiatric services are strained or inaccessible.

## Introduction

Widespread exposure to trauma and the high prevalence of post-traumatic stress symptoms (PTSD) create urgent public-health demands, especially during crises when large populations must be reached quickly. In large-scale emergencies, such as mass disasters or terror attacks, the public health challenge intensifies: mental-health services must be deployed rapidly to vast, heterogeneous populations (1). PTSD is the most widely researched consequence of such events, representing a form of post-traumatic psychopathology (2) that imposes a substantial burden on emotional well-being, interpersonal relationships, and productivity (3), making it a serious public health concern (4).

Although evidence-based treatments for PTSD are available, access is limited, and many individuals remain untreated (5). Many others experience significant symptoms without meeting the full diagnostic criteria, commonly referred to as partial, subclinical, or subthreshold PTSD (4). Additional barriers include cost, social stigma, and a shortage of mental-health professionals. Compounding these obstacles is low mental-health literacy, the “knowledge and beliefs about mental disorders which aid their recognition, management, or prevention” (6). When mental-health literacy is limited, stigma surrounding symptoms or help-seeking can further prevent service adoption (7, 8).

In this context, mental health smartphone applications (apps), such as the PTSD-Coach app, can bridge the gap by providing psychoeducational information and evidence-based cognitive behavioral tools for managing PTSD symptoms (5, 9, 10).

These apps promise scalable reach, but still adoption is not guaranteed; the effectiveness of such digital interventions hinges on users' ability to recognize the apps' value, perceive practical utility and maintain engagement long enough for full adoption. Studies have mapped different factors that influence users' adoption and continuous use of mHealth services (11), including attitude, technology anxiety, trust, technical features and design, and privacy concerns (12) or the individual cognitive factors and self-efficacy (13).

However, less is known about the pre-engagement phase, the stage in which individuals have not yet formed an intention to try the app. This gap is especially consequential for trauma/PTSD interventions after large-scale emergencies, where access barriers are high and no clinic or health organization is actively guiding individuals. Simply “informing” the public is insufficient: adherence and engagement are fragile even with clinician referrals (14). Thus, a central question emerges: How do mental health mHealth users' intentions to adopt arise in the aftermath of large-scale emergencies?.

This study examines the determinants of intention to adopt the PTSD-Coach app during the pre-engagement phase and in times of emergency.

### Study context

The study was conducted in Israel between November and December 2024, in the aftermath of the Hamas terror attack on October 7, 2023. The October 7 attack, and the subsequent Iron Swords War, inflicted widespread and unprecedented trauma on the civilian population. Communities were exposed to life-threatening violence, mass abductions, and prolonged rocket fires. Many individuals endured constant fear, repeated sheltering, forced displacement, and loss of loved ones.

National surveys conducted in April 2024 revealed that approximately 38% of Israelis reported moderate to severe symptoms of PTSD, depression, or anxiety. It was estimated that up to 900,000 adults were affected yet had not sought treatment, underscoring a massive, unmet mental health burden. Despite having a legal right to mental health care, fewer than 1% of those affected received support during the six months following the attack—a systemic failure in the midst of collective trauma (State Comptroller of Israel report, 2025).^1^.

Israel has long contended with recurrent exposure to conflict and trauma (15). Collective memory of the Holocaust continues to shape intergenerational experiences of trauma, with survivors and their descendants carrying enduring psychological and social trauma and PTSD effects (16).Yet the October 7 attack was described as unprecedented in scale and impact. Unlike previous waves of trauma exposure, the October 7 attacks and ongoing war generated a national mental health crisis that extended far beyond frontline communities. As Neria et al. (17) note, the attack triggered a profound collective trauma in Israel, reshaping public mental health needs and further exposing systemic gaps in emergency psychosocial care. Following the attack, the prevalences of probable PTSD, Subjective Traumatic Outlook (STO) depression, and anxiety were found to be high (18). With 29% for PTSD, 42%–44% for depression and generalized anxiety disorder (19), almost doubling the prevalences recorded two months before the attack. Katsoty et al. (20) predicts that approximately 5.3% of the population, may develop PTSD as a result of the terrorist attack and the war.

Moreover, since intensive media exposure to the mass trauma has been shown to aggravate PTSD symptoms and impede recovery (21), amplifying the psychological impact and extending its reach through widespread, even global, exposure to the event.

In response to the growing need for scalable interventions, the Israeli Ministry of Defense launched a Hebrew version of the PTSD-Coach app in June 2024.^2^ Adapted from the original developed by the U.S. Department of Veterans Affairs (VA) and Department of Defense (DoD), this app aims to facilitate self-management of PTSD symptoms for the general population. The app includes psychoeducational resources, self-assessment tools, evidence-based coping strategies, and access to emergency support services. The launch was accompanied by a public awareness campaign in social media.^3^.

This moment represents a rare and urgent window for studying digital mental health adoption. A time of acute collective trauma, before individuals have formally acknowledged a personal need or sought professional care. In such crisis contexts, public health systems must rapidly reach affected populations at scale, yet traditional care pathways are overwhelmed or inaccessible.

This pre-engagement phase, in which users have not yet identified their symptoms as trauma-related or initiated help-seeking, is especially critical. Individuals must first interpret their emotional responses, recognize symptom relevance, and evaluate the app's personal fit, often without clinical prompting or guidance. Understanding what drives intention in this early phase offers insight into the very first steps of digital health uptake. By examining intention formation during this unmediated period, the study addresses a key gap in mHealth adoption research and contributes evidence to support public transitions from unawareness to active self-management in the aftermath of mass trauma.

### Hypotheses development based on the literature review

Patient-centered care places individuals at the core of their own care models, and Service-Dominant Logic (S-D) parallels this stance by viewing the user as a co-creator and ultimate determiner of value (22, 23). In S-D, value emerges when actors integrate their “operant resources”, their own knowledge, skills, confidence, and networks, through activities occur across public, and private spheres (24). Building on this premise, this study argues that intention forms only when these resources are mobilized, not merely when a technological system is judged useful or easy to use.

However, most mHealth studies explain uptake through the Unified Theory of Acceptance and Use of Technology (UTAUT) and related Technology Acceptance Model (TAM) variables such as, effort and performance expectancy, ubiquity, trust, and privacy, treating intention as the proximal translation of such system perceptions into behavior (13, 25, 26). Intention functions as the mechanism through which personal assessments of usefulness and ease translate into behavioral adoption (27, 28).

While indispensable, this lens overlooks the readiness work by the user. Sweeney, Danaher, and McColl-Kennedy (29), show that “customer effort” in value co-creation improves quality of life and behavioral intentions, underscoring that users' own resources and actions are fundamental. Mensah, Zeng and Mwakapesa (30) extend the UTAUT by showing that mobile self-efficacy and intention to recommend function alongside (and sometimes instead of) classic expectancy beliefs, highlighting user capability as a primary driver. In other words, the motivational readiness that becomes intention is powered less by expectancy beliefs and more by users' confidence that they can successfully use and advocate for the service.

Sweeney, Danaher, and McColl-Kennedy (29) likewise introduce a hierarchy of customer effort in value co-creation, demonstrating that higher-effort, psychologically demanding activities are crucial for outcomes. In digital mental health tools, especially PTSD apps, low mental health literacy and self-stigma (8) can prevent intention before usability is evaluated; in trauma contexts where users may be shocked or depleted, recognizing need and mustering confidence are themselves effortful psychologically operant acts.

Therefore, this study centers trauma literacy, what users already know about trauma/PTSD causes, symptoms, and coping options, as the key upstream variable, with the expectation that higher literacy will help users perceive personal relevance and decode psychoeducational content. Using these literacy scores, this study examines intention to use in a pre-engagement phase, where no clinical referral or organizational endorsement is present, so adoption must emerge from the individual's own resources. Thus, the value creation must therefore originate in the user's operant resources (31). In that sense, value is not something the PTSD-Coach app “delivers” on its own but as something co-created through the users' intangible operant resources, their intangible capacities of knowledge, confidence, and social ties. Within S-D, operant resources mediate the passage from a product's technical attributes to a user's perceived value: users interpret, adapt, and amplify those attributes according to what they already know and can do.

Building on this evidence, this study operationalizes intention in the PTSD-Coach app context through two operant resources: self-efficacy and citizenship.

Self-efficacy was positively associated with mHealth adoption intentions (32). It acts as an internal engine of intention (33). Self-efficacy can strengthen or even substitute for weak useful beliefs (30). Mobile self-efficacy is an indicator of belief or confidence in one's skills or abilities to undertake or complete a particular course of action, such as the use of mobile health services (34). Citizenship**,** is the willingness to share, recommend, and advocate (akin to “intention to recommend” by (30) which captures the outward, social expression of intention and is shaped by social influence, advocacy, and the inverse effects of stigma, privacy concern, and perceived risk.

This study argues that operant resources, such as trauma literacy, self-efficacy, and citizenship behaviors form together a sequential path that leads to adoption intention. Thus, value is realized only when these operant resources bridge the gap between the user's lived experience and resources to the app's technical capabilities, illustrating how internal motivation and external social context jointly shape early mHealth adoption.

The research question is: In what way do users' operant resources (knowledge, self-efficacy, citizenship) most strongly predict intention to adopt PTSD-Coach in times of emergency? The hypotheses are:

**H1:** Trauma literacy has a positive effect on self-efficacy and citizenship.
**H2:** Self-efficacy has a positive effect on citizenship.
**H3**: The effect of trauma literacy on intention is mediated sequentially by self-efficacy and citizenship.
**H4**: Self efficacy is mediating trauma literacy and citizenship.
**H5:** Prior familiarity/use of PTSD-Coach is associated with higher intention and perceived value.
**H6:** Demographic and clinical variables (gender, education, trauma exposure, PTSD diagnosis, therapy status) do not significantly predict intention once self-efficacy and citizenship are included.
**H7:** The direct effect of trauma literacy on intention becomes non-significant when self-efficacy and citizenship are entered.

## Method

### The PTSD-Coach app

The PTSD-Coach app, developed by the VA/DoD, provides psychoeducation, symptom assessments, and evidence-informed self-management tools such as relaxation techniques and grounding exercises (5, 10). Designed to improve mental health literacy and coping strategies without replacing professional care, it is freely available on iOS and Android marketplaces, thereby expanding access to PTSD-related resources.

Most existing research on the PTSD-Coach app has focused on evaluating its clinical efficacy through randomized controlled trials. Kuhn et al. (5) demonstrated that at posttreatment the app led to significant improvements in PTSD, depression, and psychosocial functioning compared to a waitlist control. Similarly, Possemato et al. (9) found that clinician-supported use of the app among veterans “resulted in more improvement in patient-reported PTSD severity, higher rates of engagement in mental health treatment during the intervention period, more access to new episodes of treatment, and higher treatment satisfaction” (9).

### Study design and data collection

This study followed a mixed-methods design conducted in two sequential phases. **Phase 1** included a pre-engagement online survey, where participants were not required to download the app. The questionnaire was piloted with five individuals of varying ages, professions, and education levels. Their feedback led to revisions in wording and structure to enhance clarity. The final version was then distributed online. **Phase 2** involved two focus groups (*N* = 12) with participants who had downloaded and used the app for at least one month. However, the findings from Phase 2 are beyond the scope of this article and will not be discussed here.

#### Participants and recruitment process

Recruitment was conducted through social media advertisements, through snowball sharing and mailing lists at the researchers' academic college, targeting both students and non-students. Inclusion criteria included being 18 years residing in Israel or older and proficient in Hebrew (as the app interface is in Hebrew); exclusion criteria included not owning a smartphone or being unable to provide informed consent.

After providing consent, participants (*n* = 87; Average age 36.89, SD = 16.16) completed an online socio-demographic questionnaire along with a survey measuring their trauma literacy and intention to use the PTSD-Coach app. All responses were anonymous (except for those who volunteered to participate in Phase 2 by providing an email address).

### Questionnaire items

The research questionnaire was developed based on a comprehensive review of the relevant literature.

*Trauma literacy items* (Table 1) were adapted from the International Trauma Questionnaire (ITQ) and from the Israeli population study by Ben Ezra et al. (1). These items were not used for diagnostic purposes but rather to assess participants' mental health literacy, specifically, their knowledge and understanding of trauma and PTSD. The trauma-literacy scale showed strong internal consistency, with Cronbach's *α* = 0.84 (95% CI 0.79–0.89).

**Table 1:** The trauma literacy score. Based on your understanding and knowledge, rate (1–5) your agreement with the following statements describing PTSD symptoms.

Statement	Correct answer*	Mean (SD)
PTSD can appear shortly after the traumatic event, or months and even years later	True (4–5)	4.55 (0.78)
A person with PTSD reexperiences the traumatic event's memories	True (4–5)	4.17 (0.83)
Avoidance of thoughts/memories of the traumatic event can also lead to PTSD	True (4–5)	4.04 (1.03)
The feeling that a person is under current threat (real or not) characterizes PTSD symptoms	True (4–5)	3.99 (1.15)
Some PTSD symptoms include anger, sadness, sleep difficulties, social withdrawal, stress and anxiety	True (4–5)	4.55 (0.74)
Trauma can be collective and affect a community, group, or entire society	True (4–5)	4.19 (0.98)

* “Correct answer” refers to the direction participants should endorse to demonstrate accurate literacy.

(1–5 scale: 1 = strongly disagree, 5 = strongly agree).

*Items measuring intention to use* (Table 2) were adapted from Alves, Ferreira and Fernandes (31) and Mensah, Zeng and Mwakapesa (30) and conceptualized as a higher-order construct composed of two interrelated categories: self-efficacy, and citizenship behavior. Self-efficacy items assess belief or confidence in one's ability to cope with trauma and to use the app's tools effectively. Citizenship behavior items reflect outward-facing intentions to share, recommend, or advocate for the app.

**Table 2:** Intention to use: self-efficacy, citizenship, effort expectancy performance expectancy (1–5 scale).

Items	Category	Mean (SD)
I will feel comfortable using the app and will be able to share it with others.	Effort Expectancy	2.71 (1.29)
I believe that using the app is an important step in increasing my own and my environment's awareness of PTSD symptoms.	Performance Expectancy	?3.57 (1.29)
I believe that if I follow the recommended calming tools the app offers, my condition will improve.	Self-Efficacy	3.28 (1.19)
I believe I will be able to cope with trauma and PTSD symptoms.	Self-Efficacy	3.64 (1.15)
I will share with others the information the app provides about PTSD symptoms.	Citizenship	3.21 (1.33)
I will recommend to friends and family that they use the app.	Citizenship	3.27 (1.29)

Rate your agreement (1 = Strongly disagree, 5 = Strongly agree; 6 = Other).

In addition, two perceived value items were included to evaluate the effort expected of users in mobilizing their operant resources. The statement “*I will feel comfortable using the app and will be able to share it with others”* indexes effort expectancy, the anticipated ease and psychological comfort required to act and simultaneously signals readiness to convert self-efficacy into citizenship (sharing/advocacy). The statement “*I believe that using the app is an important step in increasing my own and my environment's awareness of PTSD symptoms”* captures performance expectancy, or perceived usefulness that extends from the self to one's network, consistent with the co-creation benefits enabled by operant resources (31). Mensah, Zeng and Mwakapesa (30) show that such expectancy beliefs translate to intention most reliably when mobile self-efficacy is present and can also directly fuel recommendation; thus, these items allow us to locate where user effort supports the activation of self-efficacy and citizenship. Together, these dimensions represent both the internal motivation and capability to act, and the external social expression of intention, yielding a multidimensional indicator of adoption intention. The intention-to-use scale demonstrated high reliability, Cronbach's *α* = 0.88 (95% CI 0.84–0.92).

*Tool preference items* (Table 3) were drawn from the actual content and features available within the PTSD Coach app (e.g., cognitive reframing, mindfulness), along with a “wish list” option (AI-based personalization) to explore user preferences for future development. These items were organized according to the same two operant resource categories used in the model: self-efficacy tools, which users can apply independently to build coping skills, and citizenship tools, which involve interaction with or support for others. Importantly, these items were not intended to constitute a unidimensional psychometric scale, but rather to capture heterogeneous, feature-specific preferences that may coexist within the same individual. Selecting self-efficacy tools signals personal confidence and readiness to act, while choosing sharing- or AI-supported tools reflects openness to co-creation and social diffusion.

**Table 3:** Tool preferences the app offers a variety of tools for coping with different symptoms. Mark what tools you would choose to use in the app.

Tool type	Category	Yes, I would use	No, I would not use
		Number, percentage	Number, percentage
Tools for cognitive reframing (changing thoughts/perspective about experiences and memories)	Self-Efficacy	63 (72.4%)	37 (42.5%)
Tools for meditation, guided imagery, mindfulness	Self-Efficacy	61 (70.1%)	39 (44.8%)
Tools to improve my self-efficacy in coping with trauma and PTSD symptoms	Self-Efficacy	68 (78.1%)	32 (36.7%)
Tools that require sharing with others (contacts or support professionals)	Citizenship	23 (26.4%)	77 (88.5%)
Tools that incorporate AI for personalized content and treatment*	Citizenship	48 (55.1%)	52 (59.7%)

* The AI-personalization option is not currently implemented in the app; it was included to measure receptivity to such a feature.

The Cronbach's alpha for the Tool Preference items (Table 3) was relatively low (Cronbach's *α* = 0.44 (95% CI 0.14–0.63). This level of internal consistency is theoretically expected given the nature of “preference” as a non-latent, multidimensional construct. Users may reasonably express interest in certain tools as speculative future options (e.g., AI-based personalization) while rejecting others (e.g., meditation or sharing with contacts), resulting in limited inter-item correlation. Accordingly, the Tool Preference items were treated as individual categorical indicators and analyzed descriptively, rather than aggregated into a single scale. Thus, the distribution of preferences across tool types provides valuable insight into how potential users imagine desirable features of digital mental health support in the pre-engagement phase.

### Statistical analysis

All statistical analyses were conducted using RStudio (version 4.4.1). Categorical variables were presented as frequencies and percentages while continuous variables were summarized using means and standard deviations or medians and interquartile ranges, as appropriate, based on their distribution. Composite scores for knowledge, self-efficacy, and citizenship were computed by averaging relevant questionnaire items. The internal consistency of each score was evaluated using Cronbach's alpha. Associations between PTSD knowledge and outcome scores were examined using Spearman correlation coefficients. Relationships between outcome scores and categorical demographic or clinical variables were assessed using *t*-tests, Mann–Whitney *U* tests, or ANOVA, as appropriate, according to the distribution of the data. Finally, multivariable linear regression model was fit to assess the effect of PTSD knowledge and other predictors (e.g., age, marital status, trauma exposure, app usage) on the overall outcome score. Model assumptions were evaluated using standard residual diagnostics, including assessment of linearity, normality of residuals, and homoscedasticity. Multicollinearity was assessed using variance inflation factors (VIFS). No major violations observed.

In addition, a formal mediation analysis was conducted using Hayes' PROCESS framework with 5,000 bootstrap resamples to examine the indirect effects and to test the hypothesized mediation pathway (H3 & H4).

*P*-value < 0.05 was considered significant.

## Results

### Respondents' demographic profile

The basic information about the respondents is presented in Table 4. Most participants were female (86.2%). The mean age was 36.89 years (SD = 16.16); thus, the largest share clustered in young to mid-adulthood. Over two thirds held an academic degree (Bachelor's 36.8%, Master's or higher 34.5%). A majority were single (41.4%), and most identified as Jewish (96.6%). More than half lived in the Jerusalem area (52.9%). Only 11.5% were currently serving in the IDF, none were recognized as disabled IDF veterans, and 10.3% reported a PTSD diagnosis. About one fifth (20.7%) had a chronic illness. Most were not in current therapy (62.1%), and usage of other health apps was roughly split (47.1% yes).

**Table 4:** Demographic information of the sample (*N* = 87).

Characteristic	Demographics	Frequency	Percentage
Gender	Male	12	13.8
	Female	75	86.2
Age (range)	18-30	45	51.7
	31–40	13	14.9
	41–50	8	9.2
	51–60	10	11.5
	61 and older	11	12.6
Education level	High school	17	19.5
	Postsecondary (incl. certificates)	8	9.2
	Bachelor's degree	32	36.8
	Master's degree or higher	30	34.5
Marital status	Single	36	49.4*
	Married/In a relationship	43*	41.4
	Divorced	6	6.9
	Widowed	2	2.3
	Separated	0	0
Religious affiliation	Jewish	84	96.6
	Christian	1	1.1
	Druze	0	0
	Muslim	0	0
	Other	0	0
Region of residence	South district	11	12.6
	Central district	24	27.6
	Jerusalem area	46	52.9
	North district	5	5.7
Currently, I serve in the IDF (regular, career, or reserves):	Yes	10	11.5
	No	77	88.5
I am recognized as an IDF disabled veteran:	Yes	0	0
	No	87	100
I am diagnosed with PTSD:			
	Yes	9	10.3
	No	78	89.7
I am diagnosed with a chronic illness (any type, incl. diabetes, cholesterol, hypertension, etc.):	Yes	18	20.7
	No	69	79.3
Are you currently receiving professional emotional/psychological counseling or therapy (of any kind)?	Yes (regularly/occasionally)	22	25.3
	No	54	62.1
	I have not received it, and I am interested in it	11	12.6
I usually use various health apps (fitness tracking, steps, meditation, etc.):	Yes	41	47.1
	No	46	52.9
Trauma Exposure: Indicate the level/type of trauma you experienced	I personally experienced a traumatic event	48	55.2
	I witnessed a traumatic event (including eyewitness, watching videos, hearing accounts)	17	19.5
	I did not experience trauma	22	25.3
Have you already known and are you using the “PTSD Coach” app?	Yes	4	4.6
	No	83	95.4
In the past week, did you experience any of the following symptoms? (Select the one most common symptom)	Anger	11	12.6
	Difficulty sleeping	12	13.8
	Anxiety or worry	18	20.7
	Sadness or hopelessness	12	13.8
	Difficulty concentrating	7	8.0
	Social withdrawal	6	6.9
	Reexperiencing the trauma	1	1.1
	None	20	23.0

* “Married (in a relationship” (25) + “Married” (18) combined here for brevity.

Over half (55.2%) personally experienced a traumatic event, while 19.5% had witnessed one. Familiarity with PTSD Coach was very low (4.6% yes).

The univariable analysis assessing associations between possible predictors of intention to use the app found that:

*Prior app use mattered*: respondents who already knew/used PTSD-Coach (*n* = 4) scored significantly higher on the intention score (M = 4.21 vs. M = 3.11; *p* = .028) and showed higher citizenship and self-efficacy, though these *p values* were non-significant (citizenship [M = 4.38 (SD: 0.95) vs. M = 3.17 (SD: 1.23) *p* = .058]; self-efficacy [M = 4.12 (SD: 0.85) vs. M = 3.45 (SD: 1.10) *p* = .234]. These results indicate an association between prior familiarity and higher intention and resource scores, although the small number of prior users limits statistical power.

**Figure 1:**
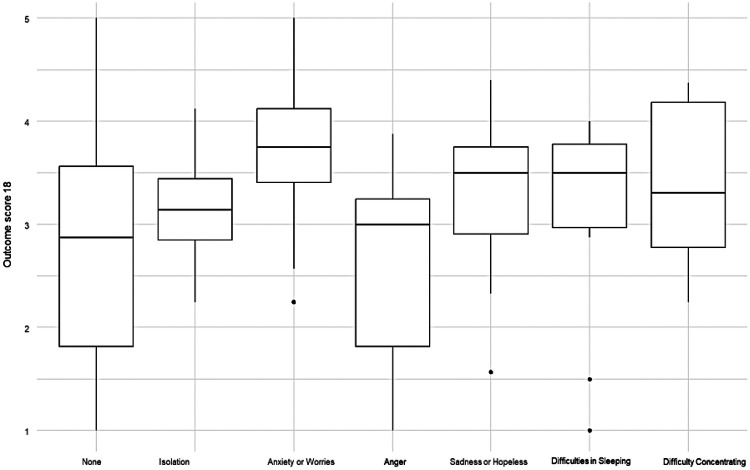
Distribution of global outcome scores by self-reported recent symptoms.

*Self-Reported Recent Symptoms*: Analysis of self-reported recent symptoms revealed variability in outcomes across groups, though formal statistical significance was limited (*p* = 0.047 for the overall outcome score, with most comparisons underpowered due to small sample sizes). As shown in Figure 1, participants who reported anxiety/worries consistently showed higher scores across domains, particularly in self-efficacy (M = 3.52, SD 1.02) and citizenship (M = 3.56, SD 0.98), suggesting that worry-related engagement may have activated stronger coping responses.

*Trauma exposure (personal vs. witnessed vs. none):* did not yield significant differences in any outcome (all *p* > .13). Thus, trauma exposure status was not associated with differences in intention, self-efficacy, or citizenship scores in this sample.

*The demographic characters:* only Family status was significant. Specifically, widowed participants (*n* = 2) had markedly lower scores on the global outcome measure (M = 1.44, SD = 0.62; *p* = .025) compared to other groups. They also scored lowest on the self-efficacy scale (M = 1.50, SD = 0.71; *p* = .070). Single participants (*n* = 36) scored M = 3.00 (SD = 1.06) on the global outcome, M = 3.04 (SD = 1.35) on citizenship, M = 3.50 (SD = 1.12) on self-efficacy. However, all other factors including Gender, education, religion, residence, army service, PTSD diagnosis, chronic illness, therapy status, and health-app use showed no significant associated with the main indices (all *p* > .05).

*The composite trauma literacy score predicted intention to use* (overall mean composite = 4.24, SD = 0.77), indicating generally high levels of trauma literacy as seen in Table 5. Demographic and clinical variables, including trauma exposure, were not associated significantly with self-efficacy, citizenship, or social capital in this pre-engagement sample. This supports this study focus on operant resources rather than fixed background traits: boosting early experience or micro-use may be more effective than targeting demographics to raise intention.

**Table 5:** Univariable prediction between knowledge and intention to use outcomes.

Comparison	Spearman's *ρ*	*P*_value	*n*
Intention to use composite score	0.316	0.004	82
Citizenship score	0.343	0.003	75
Self-efficacy score	0.358	0.001	77
Effort expectancy score	0.25	0.023	77

Based on Table 5. The study results supported **H1 H3** (The effect of trauma literacy on intention is mediated sequentially by self-efficacy and citizenship), and **H4** (Self efficacy is mediating trauma literacy and citizenship). Earlier analyses showed trauma literacy correlates with both self-efficacy and citizenship. Here, the self-efficacy items are slightly higher than the citizenship items, suggesting that knowing “what to do” (and feeling able to do it) is easier than deciding to share and recommend.

Also, **H2** (Self-efficacy has a positive effect on citizenship) is supported. The Spearman correlation results (*ρ* = .68; *p* < .001) demonstrated that higher self-efficacy strongly predicts citizenship, indicating that higher perceived capability was associated with greater willingness to share, recommend, or advocate. **H5** (Prior familiarity/use of PTSD-Coach is associated with higher intention and perceived value**), H6** (demographics/clinical factors don't matter once operant resources are in)**, H7** (The direct effect of trauma literacy on intention becomes non-significant when self-efficacy and citizenship are entered) also supported because prior app use was rare and dropped out once knowledge/self-efficacy were modeled, the table underscores that internal resources, not demographics or prior use, are carrying intention.

*The Multivariable regression model*: the Multivariable regression model in Table 6 shown here shows a linear regression including demographics (age, family status, residence, army service), prior health-app use, trauma exposure, prior PTSD-Coach use, and the composite of the trauma literacy. The study found that only trauma literacy scores significantly predicted intention to use: B = 0.42, 95% CI (0.10, 0.74), *p* = .010.

**Table 6:** The multivariable regression model.

Characteristic	Unstandardized *β* (B)	Standardized *β*	95% CI^a^ (for B)	*p*-value
Age	−0.01	−0.147	−0.03, 0.01	0.4
Marital status				
Single	—	—	—	—
Married/In a relationship	0.22	0.100	−0.37, 0.80	0.5
Married	0.41	0.176	−0.30, 1.1	0.3
Divorced	0.93	0.251	−0.15, 2.0	0.092
Widowed	−0.11	−0.012	−2.2, 2.0	>0.9
Residence				
South district	—	—	—	—
Center district	0.29	−0.012	−0.42, 1.0	0.4
North district	−0.16	−0.036	−1.3, 0.99	0.8
Jerusalem district	−0.02	−0.012	−0.73, 0.68	>0.9
Currently, I serve in the IDF (regular, career, or reserves):				
Yes	—	—	—	—
No	−0.36	−0.121	−1.0, 0.32	0.3
I usually use various health apps (fitness tracking, steps, meditation, etc.):				
Yes	—	—	—	—
No	−0.27	−0.142	−0.71, 0.16	0.2
Trauma Exposure: I witnessed a traumatic event (including eyewitness, watching videos, hearing accounts)	—	—	—	—
Trauma Exposure: I personally experienced a traumatic event	0.20	0.105	−0.39, 0.79	0.5
Trauma Exposure: I did not experience trauma	0.03	0.011	−0.66, 0.71	>0.9
Have you already known and are you using the “PTSD Coach” app?				
Yes	—	—	—	—
No	−0.69	−0.154	−1.7, 0.36	0.2
Trauma literacy score	0.42	0.2,983	0.10, 0.74	0.010

^a^95% confidence interval for the unstandardized regression coefficient (B).

All other entered covariates (e.g., age, family status, residence, army service, general health-app use, trauma-exposure types, and prior PTSD-Coach use) were not significantly associated with intention once knowledge was in the model [B = –0.69, 95% CI (−1.7, 0.36), *p* = .20; note small *n* = 4].

This model confirms the central role of trauma literacy as an upstream operant resource: it uniquely explains variance in intention after controlling for background factors. The non-significant demographics align with the proposal that fixed traits matter less than operant resources. These findings support **H1** (Trauma literacy has a positive effect on self-efficacy and citizenship) and **H6** (demographics/clinical factors don't matter once operant resources are in). In short, “what you know” about PTSD and not who you are predicts whether you intend to adopt and value the app. This suggest that what individuals know and feel capable of doing is more closely associated with adoption intention than who they are demographically.

*Self-efficacy and citizenship*: as seen in Table 7. Self-efficacy and citizenship are strongly coupled (*ρ* = 0.680, *p* < .001, *n* = 73). Users who feel more capable also report greater willingness to share, recommend, and advocate. This directly supports **H2** (self-efficacy has a positive effect on citizenship) and signals a likely mediating bridge from literacy (upstream knowledge) to outward social behaviors.

**Table 7:** Correlation between self-efficacy and citizenship (spearman).

Comparison	Correlation	*P*_value	*n*
Self-efficacy	0.680	0.000	73

Based on Table 8. In the regression model, self-efficacy is a large, significant predictor of citizenship [B = 0.73, 95% CI (0.50, 0.95), *p* < .001], whereas trauma literacy is not [B = 0.23, 95% CI (−0.11, 0.57), *p* = .20]. Once capability is accounted for, knowledge no longer explains sharing/advocacy. This pattern is consistent with **H4** (Self efficacy is mediating trauma literacy and citizenship) and **H7** (The direct effect of trauma literacy on intention becomes non-significant when self-efficacy and citizenship are entered), indicating that the effect of literacy on downstream behaviors is transmitted through self-efficacy.

**Table 8:** Model predicting citizenship score.

Characteristic	Unstandardized β (B)	Standardized β	95% CI^a^ (for B)	*P*-value
Self-efficacy score	0.73	0.62	0.50, 0.95	<0.001
Trauma literacy score	0.23	0.13	−0.11, 0.57	0.2

a CI, confidence interval.

The results of the Hayes' PROCESS analysis confirmed H3 and H4. The indirect effect of knowledge on citizenship through self-efficacy was statistically significant [ab = 0.264, 95% bootstrap CI (0.072, 0.487)]. The direct effect of knowledge on citizenship was not significant after accounting for self-efficacy (c′ = 0.237, *p* = .113). The total effect remained significant (c = 0.501, *p* = .002). The association between knowledge and citizenship was significantly mediated by self-efficacy, with the indirect effect remaining significant after bootstrapping, while the direct effect of knowledge was no longer significant once self-efficacy was included in the model.

As for H3, the results indicated that the association between trauma literacy (knowledge) and intention was fully mediated by self-efficacy and citizenship. Although the total effect of knowledge on intention was significant, the direct effect became non-significant after inclusion of the mediators. Bootstrapped analyses demonstrated a significant serial indirect effect through self-efficacy followed by citizenship [estimate = 0.132, 95% bootstrap CI (0.030, 0.287); standardized *β* = 0.079], thereby supporting H3.

To summarize, the results demonstrate a consistent pattern of associations that may fit a sequential pathway: trauma literacy relates to self-efficacy and intention (Table 5); self-efficacy tightly predicts citizenship (Table 6); and literacy's direct link to citizenship disappears when self-efficacy is included (Table 7). Together, this supports **H1**, **H2**, **H4**, and **H7**, and is consistent with the theorized path (literacy → self-efficacy → citizenship → intention).

Table 3 presents descriptive patterns of anticipated tool use within the PTSD Coach app. Preferences were consistently higher for self-efficacy–oriented tools than for citizenship-oriented tools. Most participants reported willingness to use tools for cognitive reframing (72.4%), meditation and guided imagery (70.1%), and tools explicitly designed to improve self-efficacy in coping with trauma and PTSD symptoms (78.1%), indicating broad interest in features that support personal regulation and skill-building. In contrast, willingness to use tools that require sharing with others, such as contacting support professionals or personal contacts, was relatively low (26.4%), while a large majority indicated they would not use such tools (88.5%). Openness to AI-based personalization was more mixed, with just over half of participants (55.1%) reporting willingness to use such features, despite their not currently being implemented in the app.

## Discussion

This study asked: How do intentions to adopt a PTSD mHealth app emerge, and how do users' operant resources (e.g., trauma literacy, self-efficacy, and citizenship) shape that intention? This study posited a sequential path in which the operant intangible resources translate the app's affordances into perceived value and intention to use.

*Knowledge as the upstream driver*. Consistent with **H1**, trauma literacy correlated positively with self-efficacy, citizenship, and the global intention to use score. In the multivariable model, knowledge was the only variable that significantly predicted intention, while demographics and clinical factors were null, supporting **H6**. This indicates that what people understand about PTSD is more consequential than who they are.

*Confidence converts knowledge into action*. The strong Spearman link between self-efficacy and citizenship and the regression predicting citizenship directly support **H2** and **H4**: once confidence is accounted for, knowledge no longer adds unique variance to sharing/advocacy. In other words, knowing without feeling capable does not mobilize social behavior.

*Toward a serial mediation***.** The empirical pattern supports **H3** and **H7**. Trauma literacy (knowledge) predicts intention when entered alone, but its effect drops once self-efficacy and then citizenship are added, reflecting the reduction we already observed for citizenship when self-efficacy was controlled. This is consistent with the following path: **Knowledge (trauma literacy) → Self-efficacy → Citizenship → Intention.**

Mensah et al. (30) report a parallel dynamic: “intention to use” did not automatically translate into “intention to recommend” because users lacked firsthand experience; recommendation followed from capability and actual engagement, not mere perception. In our terms, knowledge must first be converted into confidence (self-efficacy), which then enables outward advocacy (citizenship), thereby carrying the effect to intention.

In a pre-engagement emergency context, where no firm or clinician is present, users' operant resources (trauma literacy, self-efficacy, and citizenship) drive a sequential shift from “knowing” to “doing” to “advocating”: trauma literacy supplies cognitive raw material, self-efficacy converts that knowledge into willingness to act, and citizenship externalizes action through sharing and recommendation, thereby generating adoption intention. This reflects the idea that value is created through the effort users expend to integrate their own resources. Sweeney, Danaher, and McColl-Kennedy (29) label this Customer Effort in Value Co-creation Activities (EVCA), a hierarchy of increasingly demanding practices that often occur beyond the firm's boundary and show that greater effort links to better outcomes and stronger behavioral intentions. Their findings empirically support the path: once effortful, confidence-based behaviors enter the model, mere knowledge loses its direct effect.

While prior exposure to PTSD coach app was associated with higher intention in the bivariate analysis, supporting **H5**, this effect did not hold in the multivariable model. This reduction is likely attributable to the small number of prior users (*n* = 4) as well as to shared variance with trauma literacy and self-efficacy, which appear to capture overlapping aspects of preparedness for engagement. While familiarity may remain associated with motivational readiness, its independent association with adoption intention diminishes once key operant resources are considered.

Although it may be assumed that individuals who have experienced trauma would demonstrate higher trauma literacy, given their presumed familiarity with symptoms or previous exposure to care, this study found no significant association between trauma exposure and literacy scores, nor between literacy and intention to use among the trauma subgroups. This absence of association suggests that lived trauma experience does not necessarily translate into greater readiness to engage with digital mental health tools. This counterintuitive result aligns with trauma theory, which recognizes that traumatic experiences often impair cognitive processing, emotional regulation, and information uptake (35). During acute or unresolved trauma phases, individuals may experience dissociation, avoidance, or emotional numbing that may hinder their ability to recognize symptoms or to actively seek out or engage meaningfully with psychoeducational resources.

Mental health literacy, therefore, cannot be assumed to emerge organically from trauma exposure alone. Indeed, stigma, denial, and cultural framing may prevent trauma survivors from labeling their distress as PTSD or seeking help (7, 8). Thus, despite their trauma history, these individuals may exhibit low engagement patterns similar to those with no trauma history or with poor mental health literacy.

This supports the view that trauma-informed delivery is essential: knowledge alone is insufficient without the operant resources. These operant resources are necessary to mobilize behavioral readiness, especially in crisis contexts where cognitive load and psychological vulnerability are high because trauma interferes with their operant resources, for example, they may have less confidence (self-efficacy), lower ability to make sense of content (due to emotional overload), reduced trust or motivation to act socially (citizenship). Additionally, the lack of a direct association between trauma exposure and adoption intention does not indicate reduced need, but rather a gap between psychological need and the capacity for voluntary engagement under conditions of acute stress. This finding has practical implications: during acute phases of collective trauma, reliance on self-initiated uptake of digital interventions may be insufficient, underscoring the potential importance of more proactive and institutionally supported modes of introduction and outreach.

### Theoretical implications

This study advances understanding of early digital mental-health adoption by illuminating the psychological and social processes that precede formal engagement with care systems. In the context of mass trauma, where traditional mental-health services are overwhelmed or inaccessible, individuals must initiate digital help-seeking without guidance from clinicians or institutions. Findings demonstrate that intention to adopt trauma-focused mHealth tools begins not with technology features or demographic characteristics, but with internal readiness: trauma literacy supports recognition of symptoms and relevance, self-efficacy enables mobilization of coping capacity, and willingness to recommend reflects emerging social engagement with mental-health support.

By empirically mapping a sequential process from knowledge to confidence to outward advocacy, this study highlights a distinct pre-engagement phase in which psychological and social capabilities shape access to digital care. This expands current models of technology acceptance by foregrounding trauma-related cognitive and emotional processes, illustrating that help-seeking is not merely a rational evaluation of usefulness or ease, but a layered readiness task mediated by vulnerability, uncertainty, and perceived capability. Integrating trauma theory deepens this perspective: exposure to trauma does not automatically increase recognition or action; emotional overload, avoidance, and stigma may interrupt appraisal and delay digital support-seeking.

These insights suggest that digital mental-health frameworks should explicitly incorporate psychosocial readiness factors such as, trauma literacy, self-efficacy, and socially oriented intentions, as foundational to uptake during crises. Rather than viewing mHealth adoption as a purely individual or technological decision, the findings underscore that engagement emerges through cognitive understanding, emotional capacity, and supportive social context. In emergency and high-stress environments, public-mental-health interventions may benefit from strategies that first cultivate these readiness conditions, enabling digital tools to function as meaningful points of entry into care.

### Managerial implications

The results suggest a road map indicating that adoption intention in emergencies is built as a sequence of trauma literacy → self-efficacy → citizenship → intention, rather than by demographics or exposure history. According to this roadmap, trauma literacy helps users recognize relevance, self-efficacy turns recognition into capability, and citizenship externalizes capability into sharing/recommending. Trauma literacy on its own was not sufficient; effects strengthened when confidence and willingness to share were activated. This sequence can therefore orient action in two modes: pre-engagement, when the user has not downloaded and no firm is present, and post-contact, once the app is installed and guidance is possible.

In a pre-engagement stage (no download yet, no guiding firm), recommendation process are largely irrelevant because the person has not installed the app. The public-health priority is to raise trauma literacy and cultivate self-efficacy in the population. Communications can clarify what trauma/PTSD is, how symptoms unfold over time, and why brief self-help techniques can help, while pairing information with one simple, offline coping action (e.g., a one-minute breathing or grounding routine) so that knowledge is accompanied by a felt micro-success. Such pairings can be delivered through municipal and community channels, veterans' and student services, primary care waiting rooms, SMS campaigns, and discharge packets. Because timing is uncertain, people may choose to download after literacy increases, after confidence improves, or after hearing about the app from others, the operational aim is to continue offering low-effort literacy boosts together with small mastery experiences that accumulate readiness until installation occurs.

Once the app is installed, the same pathway can be cycled as a loop inside the app's service: a very brief literacy check → a guided micro-success to elevate self-efficacy → a gentle, optional invitation to normalize use or share information (citizenship). If telemetry suggests that the users hesitate to use their operant resources (e.g., tutorial skipped, long latency to first tool use), the app can re-offer concise explanations, simpler graded tasks, or brief human/chatbot coaching; if capability increases, it can surface additional tools and later, low-stakes sharing options (e.g., anonymous forwarding, “share later”). Where organizations are involved (HMOs, hospitals, veteran agencies), a short self-efficacy screener can triage users to lighter or stronger support, but the operating logic remains consistent. Iterating this loop is expected to convert initial curiosity into confident self-management and, subsequently, into diffusion through the networks, aligning practice with the mechanism observed in the data.

### Limitations and future research

The findings reported in this study reflect associative relationships consistent with a cross-sectional, correlational design and should be interpreted accordingly. The sample was small (*N* = 86), with more female participants (86%), Jerusalem-centered (53%), and highly educated (71% with academic degrees). This composition likely inflated baseline trauma-literacy scores and restricts the findings' generalizability. Several effects did not reach statistical significance; given the sample characteristics, these null findings are likely attributable to limited statistical power rather than the absence of meaningful associations.

A *post-hoc* sensitivity power analysis for multiple regression indicated that the study had 80% power (*α* = 0.05) only to detect effects corresponding to a partial R² of approximately 0.1 for an individual predictor. As demographic variables in psychosocial and mental health research typically exert small effects (36, 37), non-significant associations for demographic factors in the present analyses may reflect limited power rather than true null effects. These findings should therefore be interpreted with appropriate caution.

The trauma-exposed subgroup was also small, reducing power to test paths and associations within that group. We measured intentions but lacked behavioral metrics such as actual installs, sustained use, or in-app activity. Future studies should integrate backend analytics or longitudinal follow-ups ups to examine whether early intentions translate into real-world engagement. Clinical outcomes were not assessed, so effectiveness cannot be inferred. Experimental and micro-randomized trial designs could test which types of prompts, such as literacy cues, self-efficacy support, or social sharing nudges, are associated with engagement under different psychological conditions, thereby refining the proposed pathway over time. The cross-sectional, self-report design introduces common-method bias and precludes causal inference; longitudinal or experimental designs approaches are recommended. Future work should use longitudinal, event-based designs that track (a) timed literacy prompts, (b) momentary self-efficacy, (c) social exposures, and (d) eventual installation and early use.

Moreover, the null associations observed among trauma-exposed participants suggest that knowledge alone without affective support is insufficient. This pattern is consistent with trauma theory and underscores the possibility that digital interventions introduced during acute phases may require more proactive, supported modes of delivery rather than relying solely on voluntary self-initiation. Future research should embed psychoeducation within emotionally supportive contexts and measure shifts in felt safety, belonging, and coping confidence, alongside cognitive indicators.

This study's findings are contextually situated within Israel, a society that has long contended with recurrent traumatic events (15) and therefore should be interpreted in light of this specific civilian and collective trauma context. This may foster a heightened public disaster awareness and preparedness (38). In Israel, public awareness is reinforced through structured disaster preparedness education across all ages, which includes raising awareness of potential hazards, developing emergency plans, and training in response techniques. Moreover, it is further embedded in everyday life through advanced technologies, such as the Iron Dome missile defense system and the IDF Home Front Command smartphone application, which issues real-time alerts so that civilians can seek shelter. These institutional and cultural infrastructures may contribute to the observed associations between trauma literacy, self-efficacy, and citizenship by supporting both individual readiness and collective responsiveness.

In contrast to the U.S. veteran context in which PTSD Coach was originally developed by the VA/DoD, the Israeli setting involves widespread civilian exposure to threat and strong norms of mutual responsibility during wartime. This context may have amplified the salience of citizenship-oriented motivations, such as willingness to share or recommend the app to others, thereby shaping the observed association between citizenship and adoption intention. Accordingly, the prominence of citizenship in the present model may partly reflect context-specific dynamics associated with heightened social solidarity during crisis conditions. At the same time, citizenship can be conceptualized more broadly as a social mechanism through which individuals translate personal coping resources into collective action, suggesting that this pathway may retain relevance beyond the Israeli context. The data for this study were collected during the post–October 7 war period, amid a prolonged national emergency and elevated social cohesion, which likely influenced participants' perceptions, motivations, and reported intentions. Replication in non-conflict settings, more individualistic societies, or healthcare systems with different institutional structures will be essential to clarify the extent to which these associative patterns, particularly the role of citizenship, reflect universal vs. context-dependent processes. It is also important to recognize that patterns of adoption observed during periods of acute emergency may differ from those emerging in routine, non-crisis conditions; in such moments, “abnormal” pathways of engagement may surface regardless of cultural context, whereas different configurations of resources and motivations may shape adoption in Israel and elsewhere during times of relative stability.

Finally, While the present model foregrounds users' internal operant resources in shaping pre-engagement adoption intention, consistent with Service-Dominant Logic, it is important to acknowledge that anticipated product qualities may also function as enabling resources at this early stage. Even prior to direct use, expectations regarding usability, personalization, perceived intelligence, or features such as AI-based guidance and gamified elements may lower the threshold for initial engagement by reducing perceived effort or uncertainty. From an S-D Logic perspective, these design characteristics can be understood as value propositions whose relevance and effectiveness depend on users' interpretive and psychological resources. In this sense, product-driven affordances and user-driven resources are complementary rather than competing: while appealing design and anticipated user experience may invite exploration, trauma literacy, self-efficacy, and citizenship shape whether such affordances are perceived as usable, trustworthy, and meaningful. Future research should explicitly examine how anticipated product qualities interact with internal operant resources across different stages of digital mental health adoption, as a combined user-resource-driven and product-driven perspective may offer a more comprehensive account of pre-engagement dynamics.

## Conclusion

Intentions to adopt PTSD Coach in the pre-engagement phase emerge through a sequential path of operant resources, from trauma literacy, self-efficacy, and citizenship, rather than from demographics or mere exposure to trauma. Knowledge helps users see relevance, but only when translated into confidence to act does it drive outward prosocial behaviors like sharing and recommending.

In crisis contexts where no clinic or firm actively guides uptake, value formation begins inside the user; apps that only inform will underperform. Effective deployment must therefore (1) elevate accurate literacy, (2) engineer self-efficacy via guided action and micro-successes, and (3) catalyze citizenship by lowering stigma/privacy costs and legitimizing advocacy. Conceptually, the study emphasis the readiness stage where intention, not usage, is the primary locus of co-creation. Practically, it signals that trauma-informed, privacy-sensitive, and socially enabled designs are essential for rapid, scalable mental-health support in emergencies.

## Data Availability

The datasets presented in this article are not readily available due to confidentiality considerations. Requests to access the datasets should be directed to Keren Mazuz, kerenma@jmc.ac.il.

## Footnotes

^1^State Comptroller of Israel. (2025). *Following the October 7th attack and the Iron Swords War* [Audit report]. Office of the State Comptroller of Israel. https://library.mevaker.gov.il/sites/DigitalLibrary/Documents/2025/Swords-of-Iron-1/EN/2025-Swords-of-Iron-100-Taktzir-EN.pdf (retrieved on 24 August 2025).

^2^Based on the author’s personal communication with the PTSD-Coach project managers at the Israeli Ministry of Defense (2024–2025). They indicated that the Hebrew adaptation was initially intended for IDF veterans, modeled on the U.S. VA/DoD app, but was opened to the general public during the war to expand access (Personal communications are not publicly retrievable).

^3^As of July 2025, the Google Play Store (Android) reported over 10,000 installs of PTSD Coach. No installation data was available for the iOS version.
