# Supplementary material for: The intention to adopt mental mHealth services in emergencies: pre-engagement social determinants of PTSD-Coach app use
Source: Front Digit Health. 2026 Jan 20;8:1737779. doi: 10.3389/fdgth.2026.1737779 (PMC12864388; doi:10.3389/fdgth.2026.1737779)
Supplement: Supplementary file 1 [file Datasheet1.pdf]

Supplementary Material: The Full Survey Instrument in Hebrew

Section 1: Sociodemographic Questions

**Gender**	Male
	Female
	Other
**Age (range)**	18-30
	31-40
	41-50
	51-60
	61 and older
**Education level**	High school
	Postsecondary (incl. certificates)
	Bachelor's degree
	Master's degree or higher
**Marital status**	Single
	Married / In a relationship
	Divorced
	Widowed
	Separated
**Religious affiliation**	Jewish
	Christian
	Druze
	Muslim
	Other
**Region of residence**	South district
	Central district
	Jerusalem area
	North district
**Currently, I serve in the IDF (regular, career, or reserves):**	Yes
	No
**I am recognized as an IDF disabled veteran:**	Yes
	No
**I am diagnosed with PTSD:**	Yes
	No
**I am diagnosed with a chronic illness (any type, incl. diabetes, cholesterol, hypertension, etc.):**	Yes
	No
**Are you currently receiving professional emotional/psychological counseling or therapy (of any kind)?**	Yes (regularly / occasionally)
	No
	I have not received it, and I am interested in it
**I usually use various health apps (fitness tracking, steps, meditation, etc.):**	Yes
	No

**Trauma Exposure: Indicate the level/type of trauma you experienced**	I personally experienced a traumatic event
	I witnessed a traumatic event (including eyewitness, watching videos, hearing accounts)
	I did not experience trauma
**Have you already known and are you using the "PTSD Coach" app?**	Yes
	No
**In the past week, did you experience any of the following symptoms? (Select the one most common symptom)**	
	Anger
	Difficulty sleeping
	Anxiety or worry
	Sadness or hopelessness
	Difficulty concentrating
	Social withdrawal
	Reexperiencing the trauma
	None

Section 2: Assessment of Trauma Literacy Score

Based on your understanding and knowledge, rate (1–5) your agreement with the following statements describing PTSD symptoms. (1–5 scale: 1 = strongly disagree, 5 = strongly agree; non- applicable/other).

**Statement**	**1**	**2**	**3**	**4**	**5**	**Other**
PTSD can appear shortly after the traumatic event, or months and even years later	1	2	3	4	5	Other
A person with PTSD reexperiences the traumatic event's memories	1	2	3	4	5	Other
Avoidance of thoughts/memories of the traumatic event can also lead to PTSD	1	2	3	4	5	Other
The feeling that a person is under current threat (real or not) characterizes PTSD symptoms	1	2	3	4	5	Other
Some PTSD symptoms include anger, sadness, sleep difficulties, social withdrawal, stress and anxiety	1	2	3	4	5	Other
Trauma can be collective and affect a community, group, or entire society	1	2	3	4	5	Other

Section 3: Assessment of Intention to use: Self-Efficacy, Citizenship, Effort Expectancy Performance Expectancy (1–5 scale). Rate your agreement (1 = Strongly disagree, 5 = Strongly agree; 6=Other)

Items	1	2	3	4	5	Other
I will feel comfortable using the app and will be able to share it with others.	1	2	3	4	5	Other
I believe that using the app is an important step in increasing my own and my environment's awareness of PTSD symptoms.	1	2	3	4	5	Other
I believe that if I follow the recommended calming tools the app offers, my condition will improve.	1	2	3	4	5	Other
I believe I will be able to cope with trauma and PTSD symptoms.	1	2	3	4	5	Other
I will share with others the information the app provides about PTSD symptoms.	1	2	3	4	5	Other
I will recommend to friends and family that they use the app.	1	2	3	4	5	Other

Section 4: Assessment of Tool Preferences the app offers a variety of tools for coping with different symptoms. Mark what tools you would choose to use in the app.

Tool type	Yes, I would use	No, I would not use
Tools for cognitive reframing (changing thoughts/perspective about experiences and memories)	Yes, I would use	No, I would not use
Tools for meditation, guided imagery, mindfulness	Yes, I would use	No, I would not use
Tools to improve my self-efficacy in coping with trauma and PTSD symptoms	Yes, I would use	No, I would not use
Tools that require sharing with others (contacts or support professionals)	Yes, I would use	No, I would not use
Tools that incorporate AI for personalized content and treatment	Yes, I would use	No, I would not use
